# Reframing the Kirkpatrick model for evaluating humanistic care training in nursing: a context-sensitive integrative perspective

**DOI:** 10.3389/fpubh.2026.1789683

**Published:** 2026-03-26

**Authors:** Wan-lu Liu, Lei Chen, Lin Zhang, Liu Yang, Mei-yan Fu

**Affiliations:** 1 Department of Nursing Care, Hongqi Hospital Affiliated to Mudanjiang Medical University, Mudanjiang, China; 2 Department of Urology-Hospital Ward, Hongqi Hospital Affiliated to Mudanjiang Medical University, Mudanjiang, China; 3 Department of Pediatric Surgery, Hongqi Hospital Affiliated to Mudanjiang Medical University, Mudanjiang, China; 4 Division of Outpatient, Hongqi Hospital Affiliated to Mudanjiang Medical University, Mudanjiang, China; 5 Department of Ophthalmology, Hongqi Hospital Affiliated to Mudanjiang Medical University, Mudanjiang, China

**Keywords:** educational evaluation, humanistic care, integrative approach, Kirkpatrick model, nursing education, perspective, training evaluation

## Abstract

This perspective article critically examines the application of the Kirkpatrick model (KM) to humanistic care training in hospital-based nursing practice. While KM is widely used as a multi-level evaluation framework, its conventional linear interpretation may be insufficient for assessing relational competencies within complex clinical systems. Drawing on contemporary educational evaluation theory and empirical nursing education research, we argue that KM should be reconceptualized as a context-sensitive and probabilistic framework rather than a deterministic causal sequence. We scrutinize methodological challenges across all four levels—reaction, learning, behavior, and results—with particular emphasis on behavioral transfer, causal attribution, contextual confounding, and the limitations of self-reported measures. To enhance methodological rigor and practical applicability, we propose minimum validity conditions for each level and introduce an operationalized evaluation matrix tailored to humanistic care training. We further discuss the integration of technology-enhanced modalities, including simulation and virtual reality, as tools for strengthening multi-level evaluation. By reframing KM through an integrative health sciences lens, this perspective article provides structured, actionable guidance for designing and interpreting evaluation strategies in real-world nursing environments.

## 1 Introduction

Humanistic care is now a central focus in contemporary nursing practice, especially given increasing clinical complexity and higher expectations for patient-centered care (1). Beyond technical skills, humanistic nursing emphasizes sensitivity to patients’ emotional, cultural, and social needs, which enhances patient satisfaction, engagement, and overall care quality (1, 2). Consequently, developing and evaluating training that fosters humanistic competencies in nursing professionals has become a key concern in public health education.

Educational evaluation frameworks offer a theoretical basis for systematically analyzing the design, implementation, and practical application of training programs. Among these, the Kirkpatrick model (KM), introduced by Donald Kirkpatrick in 1959, is widely used in healthcare education (3). It conceptualizes training outcomes across four levels: reaction, learning, behavior, and results (4, 5). Rather than focusing only on immediate learner responses, KM offers a structured approach to examine both short-term educational processes and their long-term impact on practice.

Examining the application of the KM to humanistic care training in nursing is valuable from both scholarly and practical perspectives (3, 4). Conceptually, the model provides a multi-dimensional evaluation framework that helps systematically relate educational outcomes across different levels, enabling a more nuanced interpretation of training design and implementation (6–8). Practically, applying KM can clarify how humanistic care training influences clinical practice, supporting the ongoing improvement of nursing education and professional development strategies to enhance care quality and patient experience (3, 4).

From an integrative health sciences viewpoint, applying KM to humanistic care training bridges educational evaluation science with applied nursing practice (9, 10). This integration is not only technical but also conceptual, requiring careful alignment of abstract evaluation constructs with real-world clinical contexts (11). Accordingly, this perspective article discusses the application of the KM to humanistic care training in nursing, emphasizing its integrative value, methodological challenges, and implications for advancing evaluation practices in public health education.

This perspective article therefore focuses specifically on continuing professional development and in-service training in humanistic care within hospital-based nursing practice, as distinct from undergraduate education or isolated simulation exercises. By delimiting the scope to real-world clinical settings, it concentrates on contexts where challenges related to behavioral transfer, organizational integration, and causal attribution are most pronounced.

The novel contribution of this perspective article rests on three interrelated advances. First, it advances a debate-driven thesis: when applied to relational competencies, the KM should be reconceptualized from a linear evaluative ladder into a context-sensitive, probabilistic framework. Second, it proposes minimum methodological validity conditions across all four KM levels to strengthen interpretive rigor and reduce risks of overattribution. Third, it presents an operationalized evaluation matrix tailored specifically to humanistic care training, thereby extending practical applicability beyond conceptual discussion.

This perspective article argues that the KM provides a valuable multi-level structure for evaluation. However, its conventional application—typically linear and outcome-driven—is insufficient for assessing humanistic care training within complex nursing systems. We contend that meaningful evaluation should be contextually embedded and methodologically adaptive. It should also include explicit strategies to address attribution challenges, support behavioral transfer, and account for cultural influences. By reframing the KM through an integrative health sciences lens, this perspective article proposes a context-sensitive and operationalized approach designed specifically for evaluating humanistic care training in nursing practice.

## 2 Overview of the KM: from structural framework to contextual challenge

The KM provides a structured approach to training evaluation through four analytically linked levels—reaction, learning, behavior, and results—forming a multilayered framework that connects educational processes with organizational outcomes (3–5, 12). Rather than restating its well-established definitions, this perspective article focuses on how the model functions—and where it encounters limitations—when specifically applied to humanistic care training in clinical nursing practice.

The critical analysis presented in this perspective article is grounded in a structured conceptual examination of the KM’s underlying assumptions, informed by contemporary educational evaluation theory and empirical studies of its application in nursing education. Rather than relying solely on expert opinion, this analysis synthesizes published evidence from KM-based evaluations and identifies recurring methodological patterns, such as overreliance on lower-level indicators and limited longitudinal assessment of behavioral change (13–15). The interpretive framework also draws on integrative health sciences principles that emphasize contextual embedding, systems interaction, and probabilistic reasoning within complex clinical environments (6, 16).

Within this domain, each level poses distinct conceptual and methodological challenges. At Level 1 (Reaction), positive reactions may reflect alignment with professional values rather than genuine engagement with transformative content (17). At Level 2 (Learning), measuring shifts in empathy or ethical sensitivity raises concerns about instrument validity and social desirability bias (18, 19). At Level 3 (Behavior), the transfer of learning into behavior is significantly shaped by organizational culture, workload demands, and leadership support, complicating assumptions of a linear progression from knowledge acquisition to practice change (20). At Level 4 (Results), attributing improvements in patient experience or care quality directly to training is methodologically fraught due to systemic confounders and the influence of multiple teams (21).

This reinterpretation directly challenges the linearity assumption implicit in many conventional applications of the KM. In practice, the model is frequently operationalized as a sequential causal chain in which favorable reactions generate learning, learning produces behavioral change, and behavioral change culminates in organizational results (22, 23). However, within real-world nursing environments characterized by structural constraints and dynamic institutional forces, such deterministic progression is rarely empirically verifiable (20, 24). Behavioral enactment is not solely an outcome of instructional input but is contingent upon contextual moderators—including workload intensity, leadership climate, institutional culture, and staffing ratios—that may either facilitate or inhibit the integration of humanistic competencies into routine practice (25).

Moreover, causal attribution presents a particularly salient challenge at Levels 3 and 4. Observable shifts in communication behavior or patient-reported outcomes may coincide with concurrent institutional reforms, quality improvement initiatives, technological upgrades, or policy changes (26). Without systematic identification and transparent reporting of these contextual variables, evaluation risks conflating correlation with causation (27). Therefore, KM-based assessment of humanistic care training should move beyond outcome enumeration and incorporate explicit contextual mapping, confounder acknowledgment, and cautious interpretation of causal claims (11, 26).

**Figure 1:**
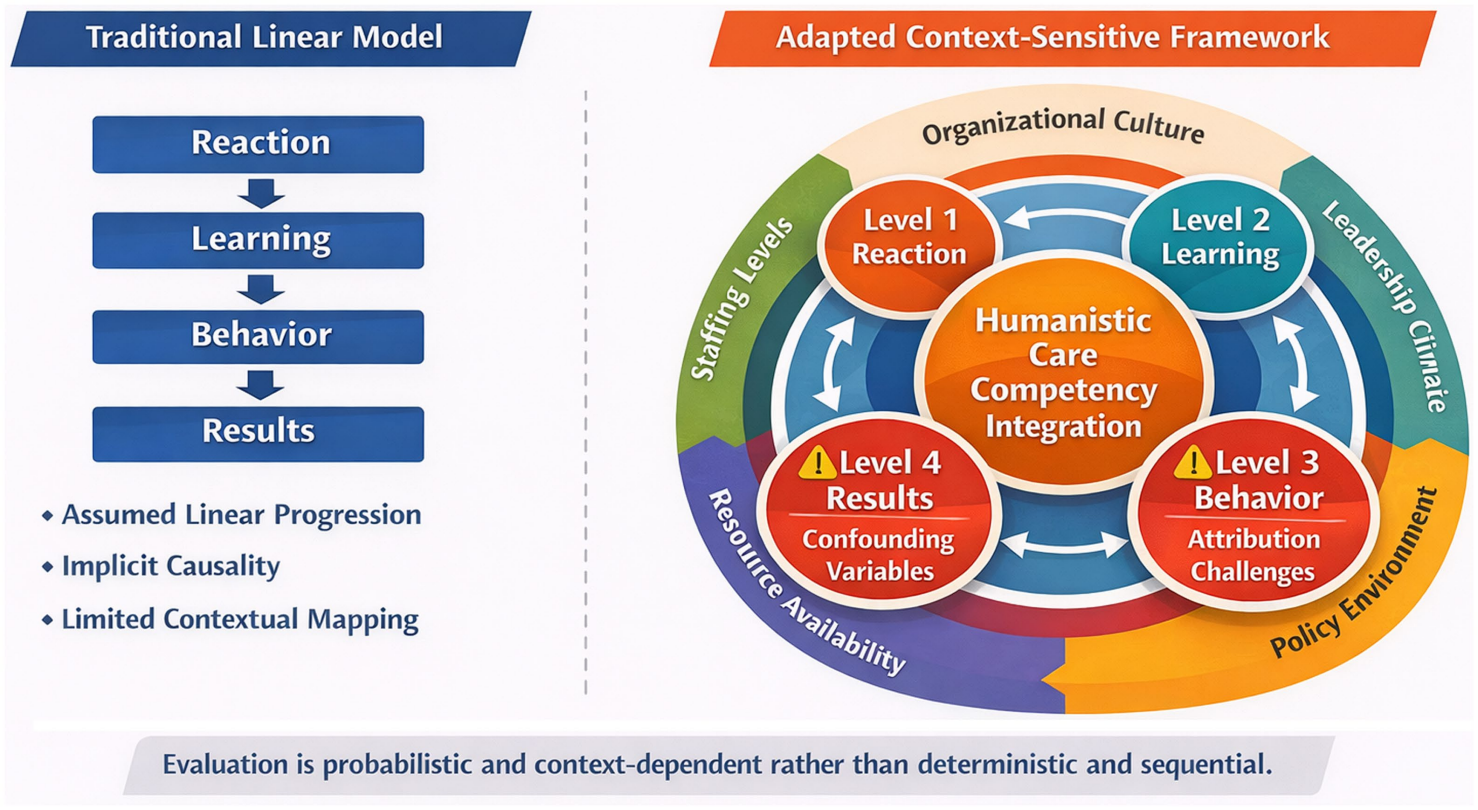
Reframing the Kirkpatrick model for humanistic care training: from linear causality to context-sensitive evaluation architecture.

Thus, while the KM offers a valuable structural scaffold, its application to humanistic care evaluation demands contextual adaptation, careful operationalization, and explicit strategies for addressing attribution and transfer (11, 22, 28). In this light, the model is best understood not as a linear causal chain but as a probabilistic, context-sensitive framework for guiding evaluation design and interpretation. Figure 1 illustrates this shift from linear causality to a context-sensitive evaluation architecture.

## 3 Current status and challenges of humanistic care training in nursing

Humanistic care training is now widely integrated into nursing education to enhance empathy, communication, ethical awareness, and patient-centered practice (7, 8, 29). Educationally, this reflects a growing acknowledgment that technical skills alone cannot meet the complex psychosocial demands of modern healthcare. However, when evaluated through a holistic framework, several structural challenges emerge.

### 3.1 Training content and pedagogical approaches

Current humanistic care training typically focuses on interpersonal communication, empathic engagement, ethical decision-making, and cultural competence (30, 31). These competencies are generally delivered through interactive workshops, role-playing, case discussions, and simulation-based learning (31). Although these methods are pedagogically sound, their evaluation often remains focused on the Reaction and Learning levels of the KM, centering on participant satisfaction and short-term changes in knowledge or attitudes (3, 5).

From an integrative perspective, this evaluation pattern reveals a disconnect between instructional design and assessment depth. While training content may align well with humanistic objectives, it is often insufficiently linked to mechanisms that promote sustained behavioral change and organizational integration (12).

### 3.2 Methodological and structural challenges

A persistent challenge in humanistic care training is the limited translation of learning into consistent clinical behavior (32, 33). Viewed through the KM framework, this issue reflects a tension between the Learning and Behavior levels. Factors such as time constraints, organizational culture, and inadequate reinforcement mechanisms can hinder the application of training in practice (33, 34).

Furthermore, many healthcare institutions lack longitudinal, multi-level evaluation systems capable of assessing outcomes beyond immediate learner reactions (8). Resource limitations and methodological complexity often confine evaluation to surface-level indicators, thereby underutilizing the integrative capacity of the KM (13). Consequently, the connection between educational activities, professional behavior, and organizational outcomes is widely recognized conceptually yet remains empirically underdeveloped (5).

Collectively, these challenges highlight the need for evaluation approaches that intentionally integrate educational theory with clinical realities, rather than treating training assessment as an isolated or episodic activity.

## 4 Critical appraisal of current applications of KM in humanistic care training

Although numerous studies report applying the KM in nursing education, closer examination reveals important methodological imbalances that support our argument for contextual adaptation. Recent publications in nursing education and training evaluation further confirm the widespread application of KM while simultaneously identifying persistent limitations in higher-level outcome measurement and longitudinal design (15, 35–37). These contemporary findings underscore the necessity of critically reassessing methodological rigor and contextual adaptation in current evaluation practice, particularly when addressing relational competencies such as empathy and communication (15, 37).

For instance, Rasouli et al. (7) evaluated virtual nursing training using KM but limited assessment primarily to Levels 1 and 2, relying on post-training satisfaction surveys and knowledge tests without longitudinal behavioral follow-up. Similarly, Lee and Song (8) conducted a scoping review of accelerated nursing programs and found that most included studies reported reaction and short-term learning outcomes, whereas very few assessed behavioral transfer or organizational impact.

By contrast, Miranda et al. (13) identified that only a minority of nurse training studies implemented all four KM levels comprehensively. Even among studies claiming Level 3 evaluation, behavioral measurement often consisted of short-term self-reports rather than structured workplace observations (13). This pattern recurs: higher-level outcomes are either omitted or operationalized indirectly (38).

Additional empirical evidence from compassion-focused and communication-based nursing education further illustrates these methodological tendencies. Adamson and Dewar (29) documented reflective growth following narrative-based compassionate care training; however, they did not evaluate whether such attitudinal changes were sustained in routine clinical practice over time. Similarly, Beach et al. (39) reported short-term improvements in culturally responsive communication after educational interventions, yet found limited evidence linking these gains to measurable patient-level outcomes. Together, these findings underscore a recurring evaluative gap: while affective or cognitive shifts are often documented, longitudinal behavioral enactment and organizational impact remain insufficiently examined (40, 41). This reinforces the necessity of multi-level, longitudinal evaluation designs that extend beyond attitudinal measurement to capture durable practice transformation and system-level effects.

These inconsistencies are not merely technical shortcomings; rather, they reflect structural challenges inherent in evaluating relational competencies. Humanistic care training aims to influence empathy, communication, and ethical responsiveness—domains that are inherently context-sensitive and difficult to quantify (42, 43). Consequently, many studies default to Level 1–2 indicators due to feasibility constraints (11, 22).

Moreover, causal attribution at Level 4 remains underdeveloped (22). Improvements in patient satisfaction or care quality are frequently reported without controlling for confounding factors such as staffing ratios, leadership climate, or concurrent quality initiatives (44). Without appropriate contextual adjustment, claims regarding organizational “results” risk overinterpretation.

These patterns substantiate our central thesis: the issue is not insufficient application of KM per se, but insufficient methodological adaptation to the complexity of humanistic care within real-world nursing systems.

## 5 Advantages and limitations of the KM from an integrative perspective

From the perspective of integrative health sciences, the main strength of the KM lies in its ability to connect educational processes with considerations at the practice and organizational levels (9, 13). By conceptualizing evaluation as a multi-level continuum, KM encourages stakeholders to move beyond isolated metrics and consider how learning is embedded within broader healthcare systems (9).

A notable advantage of the model is its conceptual clarity. The four-level structure provides a common evaluative language that can be shared across educational, clinical, and managerial domains (5, 13). This is especially valuable in the context of humanistic care training, where outcomes are multidimensional and cannot be easily captured by a single metric. For example, training in communication skills not only influences participants’ immediate reactions and learning but also affects long-term behaviors and patient satisfaction, highlighting the need for a multi-faceted evaluation framework (9).

Simultaneously, the application of the KM highlights significant methodological tensions. Data collection at the Reaction and Learning levels often relies on self-reported measures, which may be subject to biases such as social desirability or recall inaccuracies (22, 45). In the context of humanistic care, these vulnerabilities are amplified by cultural and professional norms that idealize compassion and empathy as core nursing virtues (46). In collectivist healthcare environments in particular, respondents may overreport empathic attitudes or communication competence to align with professional identity expectations (47, 48). Consequently, reliance on self-report instruments alone risks overstating attitudinal change and obscuring the gap between expressed values and enacted behaviors (49). To mitigate such distortion, triangulation is methodologically essential—combining validated self-report scales with patient-reported outcome measures and structured behavioral observations in authentic clinical settings.

In contrast, evaluation at the Behavior and Results levels presents a different set of challenges. These levels require sustained institutional commitment, longitudinal monitoring, and integration of multi-source data streams (10, 45). Behavioral transfer is contingent upon contextual variables—including workload, staffing ratios, leadership culture, and organizational climate—that may facilitate or constrain the application of newly acquired competencies (50). Similarly, improvements in patient satisfaction or care quality cannot be attributed solely to training interventions without careful adjustment for confounding systemic factors (51). These complexities underscore the limitations of assuming linear progression across KM levels in relational competency training.

To enhance methodological rigor and interpretive transparency in light of these challenges, we propose baseline validity conditions tailored to each level of the KM. At Level 1, anonymous data collection, inclusion of qualitative commentary, and transparent reporting of response rates are recommended to reduce inflation bias and improve data credibility (52, 53). At Level 2, assessments should employ validated instruments with reported reliability coefficients and incorporate pre–post or comparative designs to strengthen internal validity (54). At Level 3, observational evaluation should occur no earlier than 3 months post-training and utilize structured behavioral checklists alongside triangulated data sources to capture sustained practice change (53, 55). At Level 4, evaluators should explicitly identify contextual confounders and, where feasible, apply statistical adjustment or matched comparison approaches to strengthen attributional validity (56). These conditions are not intended as rigid prescriptions, but rather as minimum methodological safeguards necessary to ensure credible interpretation of evaluation findings in humanistic care contexts.

Rather than diminishing the model’s relevance, these limitations underscore the need for adaptive and context-sensitive implementation strategies. Successfully integrating KM into nursing practice requires not only methodological rigor but also alignment with organizational workflows, institutional culture, and professional development frameworks (22). In this sense, KM should not be viewed as a prescriptive checklist but rather as a flexible framework that should be interpreted and applied according to the specific context (11, 22). For instance, in settings where longitudinal tracking is not feasible, evaluators might focus on strengthening data collection at the Reaction and Learning levels while incorporating proxy indicators for Behavior and Results (10). By doing so, KM can remain a valuable tool for understanding and improving training outcomes without overburdening organizations.

The model’s flexibility thus allows it to serve as a guiding structure rather than a rigid formula, enabling educators and administrators to adapt it to varied environments while maintaining a systematic approach to evaluation (13, 57). This adaptability not only enhances the practicality of KM in real-world settings but also supports its continued relevance in advancing both educational quality and patient-centered care outcomes (14).

## 6 Future directions: toward operationalized and context-sensitive evaluation of humanistic care training

To advance evaluation practice beyond conceptual alignment, future research and implementation efforts should adopt more precise, context-sensitive strategies tailored to humanistic care competencies.

### 6.1 Operationalizing empathy and communication assessment across KM levels

At Level 2, evaluation of empathy and communication competencies should employ validated psychometric instruments—such as standardized empathy scales—alongside structured reflective writing to capture both affective and cognitive shifts (58, 59). Where feasible, pre-post designs should include comparison or control groups to mitigate maturation and testing effects (60).

At Level 3, structured behavioral observation tools should be implemented within authentic clinical encounters (61). Trained observers may, for instance, use standardized communication checklists to assess active listening, emotional acknowledgment, and patient engagement behaviors (61). Delayed follow-up (e.g., 3–6 months post-training) is recommended to evaluate sustained behavioral transfer rather than short-term compliance.

At Level 4, patient-reported measures of relational care may serve as cautiously interpreted outcome indicators (58). However, evaluation designs should incorporate adjustments for contextual confounders such as staffing ratios, leadership climate, and concurrent quality initiatives (62). Multivariate statistical modeling or matched comparison designs can enhance attribution validity.

To enhance reproducibility and implementation consistency, Table 1 presents a structured evaluation matrix that systematically aligns each KM level with corresponding humanistic care indicators, recommended data sources, appropriate timing intervals, and strategies for mitigating bias. By consolidating these methodological elements into an integrated framework, the matrix functions as a practical translation of theoretical principles into actionable guidance for evaluation design and program implementation (Table 1).

**Table 1:** Context-sensitive evaluation matrix for humanistic care training based on an adapted KM framework.

KM level	Humanistic care indicator	Data source	Timing	Common bias	Methodological adaptation
Level 1-Reaction	Perceived empathy relevance	Anonymous post-training survey	Immediate	Social desirability bias	Use anonymized digital collection
Level 2-Learning	Empathy scale score change	Pre-post validated scales	Short-term	Limited capture of dispositional change	Include reflective narrative assessment
Level 3-Behavior	Observed communication behaviors	Structured observation checklist	3–6 months	Hawthorne effect	Delayed and blinded observation
Level 4-Results	Patient-reported care experience	Standardized patient satisfaction tools	6–12 months	Attribution bias	Adjust for contextual confounders

KM, Kirkpatrick model.

Collectively, these strategies reflect a shift from abstract evaluation principles toward measurable, reproducible indicators specifically aligned with humanistic care training.

### 6.2 Integrating virtual reality and simulation into humanistic care evaluation

Technology-enhanced modalities offer promising avenues for bridging learning and behavior. Simulation-based communication training-particularly using standardized patient scenarios-has demonstrated measurable improvements in observable interpersonal behaviors (63).

Virtual reality (VR) environments may further enhance empathic perspective-taking by immersing nurses in simulated patient experiences, such as scenarios depicting sensory impairment, chronic pain, or emotional distress (10). Such immersive interventions allow controlled assessment of behavioral responses and reduce reliance on self-report measures. For example, empathy-focused VR modules can incorporate embedded performance metrics-including response latency, verbal acknowledgment patterns, and nonverbal orientation behaviors-enabling objective behavioral tracking within Level 3 evaluation (64).

Importantly, technology should not be evaluated as an isolated innovation. Rather, VR and simulation should be embedded within KM-based evaluation frameworks to systematically examine their contribution to sustained behavioral transfer and patient-centered outcomes (64, 65). This integration enhances methodological rigor while preserving contextual relevance (66).

### 6.3 Actionable recommendations for contextual adaptation

To operationalize a context-sensitive KM framework for humanistic care training, we propose several actionable recommendations. First, minimum methodological standards should be established for each KM level, including requirements for validated instruments and clearly defined follow-up intervals (22). For Level 3 evaluation specifically, triangulation across at least two data sources—such as combining supervisor observations with patient feedback or self-report—should be required to strengthen validity (67). At Level 4, systematic assessment of contextual confounders, including staffing ratios, leadership climate, and concurrent quality initiatives, should be incorporated into outcome analyses to enhance attribution accuracy (68, 69). Evaluation planning should also be aligned with institutional capacity to ensure feasibility and sustainability over time. Finally, evaluation metrics should be embedded into ongoing professional development systems rather than treated as episodic, one-time assessments (70). Collectively, these steps move beyond broad conceptual suggestions and provide a structured, implementable pathway for advancing humanistic care evaluation within diverse healthcare environments.

## 7 Discussion

This perspective article argues that while the KM remains a valuable evaluative framework for humanistic care training, its conventional linear interpretation proves inadequate for assessing relational competencies within complex nursing systems.

Throughout this perspective, we have demonstrated that the central challenge lies not in the absence of KM application, but in the lack of contextual adaptation. Humanistic care competencies—including empathy, ethical responsiveness, and patient-centered communication—are inherently relational, culturally situated, and shaped by organizational dynamics (30–32). Accordingly, evaluation should account for interactive effects across levels, contextual confounders, and the temporally extended nature of behavioral integration (9, 22, 32).

In response to these complexities, we have proposed structured data strategies, triangulated behavioral assessment methods, and context-adjusted attribution safeguards. Together, these approaches reframe KM as a probabilistic, context-sensitive evaluation architecture rather than a deterministic causal chain. Crucially, we emphasize that methodological rigor and contextual awareness should function in tandem: measurement precision without contextual interpretation risks misattribution, while contextual sensitivity without structured evaluation invites subjectivity.

To further mitigate the risk of overinterpretation, future applications of KM in humanistic care evaluation should explicitly delineate attributional boundaries and systematically report contextual variables alongside outcome findings (11, 22, 71). Clear operational definitions, predefined methodological safeguards, and structured documentation of potential confounders are essential to enhance reproducibility and facilitate meaningful cross-institutional comparison (72). Embedding these principles into evaluation design enables the model to evolve from a descriptive classificatory framework into a more rigorous interpretive instrument—one capable of addressing the epistemological and practical challenges inherent in evaluating complex relational competencies in nursing practice.

Ultimately, advancing the integration of educational evaluation science with nursing practice requires moving beyond checklist compliance toward evaluation designs that are theoretically informed, operationally transparent, and culturally responsive (11, 22, 73). Such recalibration not only strengthens the validity of humanistic care training assessment but also reinforces its credibility within broader healthcare quality improvement systems (74).

## Data Availability

The original contributions presented in the study are included in the article/supplementary material, further inquiries can be directed to the corresponding author.
